# Depression and its psychosocial risk factors in pregnant Kenyan adolescents: a cross-sectional study in a community health Centre of Nairobi

**DOI:** 10.1186/s12888-018-1706-y

**Published:** 2018-05-18

**Authors:** Judith Osok, Pius Kigamwa, Ann Vander Stoep, Keng-Yen Huang, Manasi Kumar

**Affiliations:** 1 Department of Psychiatry, School of Medicine, College of Health Sciences, University of Nairobi, P. O. Box 20386, 00100 Nairobi, Kenya; 2 Department of Psychiatry, School of Medicine, College of Health Sciences, University of Nairobi, P. O. Box 19676 (00202), Nairobi, Kenya; 3 Psychiatry & Behavioral Sciences and Epidemiology, Child Health Institute, University of Washington, 6200 NE 74th Street, Suite 210, Seattle, WA 88115-1538 USA; 4 Department of Public Health and Child and Adolescent Psychiatry, New York University, New York, NY 10016 USA; 5 Department of Psychiatry, College of Health Sciences, University of Nairobi, Nairobi, 00100 (47074) Kenya; 6 Research Department of Clinical Health and Educational Psychology, University College London, London, WC1E 7BT UK

**Keywords:** Adolescent pregnancies, Prevalence of depression, Psychosocial risk factors

## Abstract

**Background:**

Adolescent pregnancies within urban resource-deprived settlements predispose young girls to adverse mental health and psychosocial adversities, notably depression. Depression in sub-Saharan Africa is a leading contributor to years lived with disability (YLD). The study’s objective was to determine the prevalence of depression and related psychosocial risks among pregnant adolescents reporting at a maternal and child health clinic in Nairobi, Kenya.

**Methods:**

A convenient sample of 176 pregnant adolescents attending antenatal clinic in Kangemi primary healthcare health facility participated in the study. We used PHQ-9 to assess prevalence of depression. Hierarchical multivariate linear regression was performed to determine the independent predictors of depression from the psychosocial factors that were significantly associated with depression at the univariate analyses.

**Results:**

Of the 176 pregnant adolescents between ages 15-18 years sampled in the study, 32.9% (*n* = 58) tested positive for a depression diagnosis using PHQ-9 using a cut-off score of 15+. However on multivariate linear regression, after various iterations, when individual predictors using standardized beta scores were examined, having experienced a stressful life event (B = 3.27, *P = 0.001*, β =0.25) explained the most variance in the care giver burden, followed by absence of social support for pregnant adolescents (B = − 2.76, *P = 0.008*, β = − 0.19), being diagnosed with HIV/AIDS (B = 3.81, *P = 0.004*, β =0.17) and being young (B = 2.46, *P = 0.038*, β =0.14).

**Conclusion:**

Depression is common among pregnant adolescents in urban resource-deprived areas of Kenya and is correlated with well-documented risk factors such as being of a younger age and being HIV positive. Interventions aimed at reducing or preventing depression in this population should target these groups and provide support to those experiencing greatest stress.

## Background

Adolescents are defined as people aged between 10 and 19 years according to the World Health Organization (WHO) [1]. Kenya is a country with an overwhelming population of children and youth, with more than half the estimated population of 36 million under the age of 18 years [2]. Two thirds of young people, worldwide are growing up in countries like Kenya where preventable and treatable health problems like HIV/AIDS, early pregnancy, unsafe sex and depression are common and where injury and violence remain a daily threat to their health, wellbeing and prospects in life [3–6]. In the African continent the rate of urbanization soared from 15% in 1960 to 40% in 2010 and is projected to reach 60% in 2050 [7]. Rapid urbanization has meant that economic growth and opportunities are centered in bigger cities and these pockets also fuel huge inequalities such as high unemployment and low incomes; inadequate housing together with tenure insecurity; and limited access to services such as health, education, safe water and sanitation, with limited physical and environmental infrastructure [8]. With the growth of urban informal settlements, populated with an emerging class of citizens known as ‘urban poor’; a large number of whom are children and young people with poor access to health, education or even basic sanitation and resources in an additional challenge in several African counties including Kenya [9]. Poor family planning, sexual and reproductive health education and services and absence of robust maternity services are common problems in these informal settlements [10]. Adolescent pregnancy is then unintended and unplanned, fueled by socioeconomic and cultural factors and their prevalence aggravated by poor dissemination of sexual and reproductive health knowledge and access to adolescent friendly health services. In the urban resource- deprived settlements it predisposes young girls to adverse mental health outcomes and enormous psychosocial stresses including stigma and discrimination [3].

### Being an adolescent: Risk factor for both perinatal health and depression

Pregnancy is ordinarily a natural developmental process however evidence shows that a significant portion of adolescent fertility is unintended–either unwanted or mistimed–across countries in SSA [9]. Adolescent pregnancy is associated with poor health outcomes, including maternal deaths and injuries and adverse infant outcomes [11]. Eleven percent of all births occur in women between 15 and 19 years [12] with lower-and-middle income countries (LMICs) accounting for 95% of births before 18 years. Almost 10% of girls in LMICs become mothers by the age of 16, with the highest prevalence rates of early pregnancies seen in Sub-Saharan Africa (SSA) and in South Central and Southeast Asian regions [13] In Kenya, 26% of adolescents become mothers between 15 to 19 years of age [14] About 41% of these pregnancies are unintended, 26% are mistimed and 15% are unwanted [9]. These unintended and early pregnancies also make adolescents prone to STIs and HIV, adolescents experiencing an unwanted pregnancy are more likely to resort to abortions, which are often illegal and done by unskilled attendants leading to several pregnancy and birth-related complications [15]. Adolescent pregnancy predisposes young girls to adverse mental health [3] with depression and anxiety being the most common mental health disorders [16, 17]. Worldwide prevalence of depression during pregnancy is estimated to be between 11 and 18% [4, 18]. A higher prevalence is reported in women in LMICs with point prevalence of 15.6% during pregnancy and 19.8% post-partum [19, 20]. Major depressive disorder (MDD) accounts for approximately 40% of YLDs yet mental health services in Sub-Saharan Africa are mostly restricted to tertiary psychiatric facilities [21–23]. Recent studies have shown that the prevalence of depression across sub-Saharan Africa to be high with antenatal depression reported to be 27 and 33% respectively in Ghana and Côte d’Ivoire [24]. In Ethiopia, the prevalence of antenatal depression was 24.94% [25] and 39.5% in Tanzania [26]. In Kenya, a longitudinal study of women attending maternal and child health clinics in two major public hospitals in Nairobi (using Kiswahili version of EPDS with a cut off of 13 or more) found a prevalence of 18% for antepartum depression [27]. Given this context, and how rampant adolescent pregnancies are in LMICs like Kenya, the clear implementation gap has been to provide timely integrated sexual and reproductive health, maternity and mental health services in these informal settlements. For this implementation gap to be addressed we need robust epidemiological studies that look at depression prevalence, its severity and the associated risk factors that aggravate mental illness in this vulnerable group.

### Depression and mental health problems in pregnant adolescents

In Kenya and other SSA countries, adolescent pregnancy is widely prevalent public health problem which poses a significant mental health burden. Despite this only a few studies on adolescent mental health needs during perinatal context can be found. Women living in LMICs are exposed to multiple risk factors for depression as their adverse life circumstances commonly include poverty and lack of family structure, low level of education, high rates of HIV/AIDS diagnosis, stigma and lack of social support [28–31]. There is a huge evidence now which suggests that the risk of depression is heightened when there is a previous adolescent pregnancy, low partner age, and adolescents whose mothers themselves had early unintended pregnancies [32, 33]. Furthermore, there is evidence of an association between a mood disorder and concomitant alcohol and substance use in adolescents in SSA which presents additional pregnancy and birth related risks [34]. One study reported that 43.7% of adolescents attending public secondary schools sample in Nairobi had clinically significant depression scores; nearly the same proportion were detected with moderate to severe anxiety along with depression symptoms [35]. Our study aims to determine the prevalence of depression in pregnant adolescents living in a resource-poor urban settlement of Nairobi County and an associated objective is to identify related psychosocial risk factors accompanying depression in this vulnerable population.

## Method

### Study design, population and setting

We carried out a cross-sectional study assessing depression and associated psychosocial risk factors in pregnant adolescents attending a Maternal Child Health clinic at a Nairobi community health care center located within the informal settlement. The facility is operated through the County Council of Nairobi giving free maternity services and caters for low-and-middle income wage earners from nearby informal settlements. It receives between 12 and 15 pregnant women every day and operates every weekday. We recruited 176 participants between ages 15-18 years using a prevalence rate of 13% from a study [36]; using Cochran sample size estimation (1977) for a cross-sectional study keeping alpha at *p* < 0.05.

### Study procedures

This study was approved by Kenyatta National Hospital and University of Nairobi Ethics Review Committee (approval no. P499/07/2015). We collected data between November, 2015 to January, 2016. The study’s purpose was explained to the participants and their caregivers. A written informed consent was signed based on willingness to participate in the study and for future uses of data, such as publication, preservation and long-term use of research data. About 6.8% (*n* = 12) of pregnant adolescents we approached during data collection declined to participate in the study and despite our assurance of anonymity and confidentiality, it was mostly due to a strong feeling of stigma associated with their pregnancy. We suspect that their partners or caregivers prohibited them from participation. Adolescent pregnancy has a significant social stigma associated with it in the community context where we were working.

Once data was collected, confidentiality was assured by other means such as by de-identifying the data by use of serial numbers and the paper data was kept in sealed boxes in the Department of Psychiatry office. The study was conducted in a small cubicle within the ANC clinic in the health facility. Participants completed the researcher-designed demographic and psychosocial risk factors questionnaire and PHQ-9 Kiswahili version. There was no incentive given for participation. The lead author, who carried out this study as part of her postgraduate research, is a trained clinical psychologist with wide experience in working with adolescents and young children in the informal settlements of Kenya.

The study team discussed the data collection, referral mechanisms and use of the tools with the lead researcher who collected all the data herself. The participants recording high scores with a cut off of 15+ on the PHQ-9 were referred to one of two places: (a) Psychiatric clinic operated on Wednesdays within the facility or (b) provided alternative of the Youth/Reproductive Health Centre at Kenyatta National Hospital where they could be seen free of charge. Within this inquiry, we had a nested small qualitative study exploring participants’ interpersonal, practical and cultural challenges and barriers to accessing depression and general mental health care. The substantive findings of this qualitative inquiry are under process [37].

### Measures

#### Research designed questionnaire

We inquired from our participants about their living conditions, access to food, care and education, whether they lived with parents, relatives or partners, income source/s, parental and partner social support and education, alcohol/substance abuse, experience of sexual/domestic violence, and STI/HIV status. These are social risk factors known to be associated with adolescent pregnancy and are also commonly found in adolescents with depression.

#### Perinatal depression screening tool

We used Kiswahili translated version of Edinburgh Postnatal Depression Screen (EPDS) [38]. EPDS has demonstrated acceptable clinical utility as a screening scale in Kenya and SSA. Our study team at the University of Nairobi has been using it to assess perinatal depression including carrying out a formal Kiswahili translation of EPDS and cross-cultural emic-etic issues in translation [39] and it was this version of the tool that was administered orally to the participants. The lead researcher first gave the socio-demographic tool followed by EPDS. A cut off of 13+ confirmed presence of peri-partum depression. A cut-off of 13 is recommended for probable major depression and a cut-off of 10 is recommended for probable minor depression [40]. We used this instrument as a primary screener and to enhance comparability with other studies carried out in Kenya.

#### Depression diagnosis and severity assessment

We used PHQ-9 as our main outcome variable (> 15+) and also to identify severity [41], the higher scores are an indication of greater severity depression.

Due to the peripartum nature of depression in adolescents, we used EPDS as a screener to identify likelihood of depression. We reported scores on PHQ- 9 for those who tested positive in EPDS primarily for test-retest reliability [42] and to categorize depression severity. The collection of data from these tools ensured internal validity through triangulation in evaluation of data and findings while external validity was obtained to the extent that these study findings can be generalized to other populations. During assessments, we targeted participants whose gestation period was 4 months and above and sought clarification on the duration of somatic symptoms of depression from normal pregnancy related symptoms. Participants who scored above > = 15+ on PHQ-9 (i.e. from moderately severe category onwards) were considered to have symptoms of depression and were therefore referred for specialized care.

### Statistical analysis

SPSS version 22 [43] was used in data analysis. The association between depression and its psychosocial correlates was determined in two ways. Firstly, we divided our sample into two groups (depressed and non-depressed according to PHQ-9 cut-off score 15+ or more) and compared these groups using *chi-square* test. Secondly, we assessed each potential correlates with the PHQ-9 score using independent samples *t-test* and ANOVA. Hierarchical multivariate linear regression analyses were performed to determine the independent predictors of depression from the psychosocial factors that were significantly associated with depression at the univariate analyses. We ran regression analysis by entering the participants’ socio-demographical variables into Block 1, followed by other characteristics/ conditions in Block 2. There were no missing data for all the independent and dependent variables. Prior to running the analysis, all assumptions were checked including univariate/multivariate normality, linearity, homoscedasticity and diagnostic testing for multi-collinearity and independence of errors. After checking for univariate normality, the PHQ depression scores was transformed by a two-step approach using inverse distribution function (IDF) using maximum likelihood estimator (MLE) in which we retained the original series mean and standard deviation to improve the interpretation of results. The level of statistical significance was kept at *P* < 0.05, all tests were two sided.

## Results

### Sample characteristics

The socio-demographic characteristics associated with depression in our sample can be found in Table 1. Over half of our sample was 18 years of age, most had had primary but not secondary level of education and about half were married. A quarter was gainfully employed and just under half reported their family income to be less than 10,000 Kenyan shillings per month (less than 100 USD per month). A quarter lived in temporary shelters or houses and a large majority (91.5%) attended the antenatal clinic as scheduled. Approximately a quarter of participants reported that they had not received any support from their partner or family members but just over half said they had received a positive response from their partners or parents once pregnancy was disclosed. A large number of our participants had experienced a recent traumatic event such as bereavement, loss of parent or close family member, abuse or feuds in their lives. A third had experienced domestic abuse and about 8 % of our participants were adolescent living with HIV.

**Table 1:** Demographic and Psychosocial Risk Factors Associated with Depression amongst respondents

Variable	Category	Overall (*N* = 176)	Non-Depressed^1^ (*N* = 118)	Depressed^2^ (*N* = 58)	Mean(SD) PHQ-Score	95% C.I.	Group Differences
Age in years	15-16 Years	27(15.3)	7(25.9)	20(74.1)	17.2(4.0)	(15.6-18.8)	(*F*(2, 173) = 18.63; ***P*** **< 0.001**)^†^
	17 Years	41(23.3)	25(61.0)	16(39.0)	11.3(7.0)	(9.0-13.5)	
	18 Years	108(61.4)	86(79.6)	22(20.4)	9.5(5.7)	(8.5-10.6)	
Occupation	Unemployed	133(75.6)	84(63.2)	49(36.8)	11.8(6.4)	(10.7-12.9)	t_(174)_ = 2.3; ***P*** **= 0.020**
	Actively employed	43(24.4)	34(79.1)	9(20.9)	9.2(5.9)	(7.3-11.0)	
Education level	Primary and below	141(80.1)	89(63.1)	52(36.9)	11.5(6.6)	(10.4-12.6)	t_(174)_ = 1.7; *P* = 0.091
	Secondary and above	35(19.9)	29(82.9)	6(17.1)	9.5(5.3)	(7.7-11.3)	
Marital status	Unmarried	85(48.3)	40(47.1)	45(52.9)	14.7(5.1)	(13.6-15.8)	t_(174)_ = 8.4; ***P*** **< 0.001**
	Married	91(51.7)	78(85.7)	13(14.3)	7.8(5.7)	(6.6-9.0)	
Living arrangements	Living with other	113(64.2)	48(58.5)	34(41.5)	8.6(5.9)	(7.5-9.7)	t_(174)_ = −8.1; ***P*** **< 0.001**
	Living with parents	63(35.8)	42(67.7)	20(32.3)	15.6(4.6)	(14.4-16.8)	
Monthly income	< 10,000Ksh.	82(46.6)	28(87.5)	4(12.5)	12.4(6.2)	(11.0-13.7)	(*F* (2, 173) = 3.42; ***P*** **= 0.035**)^†^
	10,000-14,999Ksh	62(35.2)	12(31.6)	26(68.4)	10.5(6.9)	(8.7-12.2)	
	> = 15,000Ksh	32(18.2)	106(76.8)	32(23.2)	9.2(5.0)	(7.4-11.0)	
Type of housing	Temporary	38(21.6)	93(82.3)	20(17.7)	15.2(6.1)	(13.2-17.2)	t_(174)_ = 4.7; ***P*** **< 0.001**
	Permanent	138(78.4)	25(39.7)	38(60.3)	10.0(6.0)	(9.0-11.0)	
Receiving social support	No	40(22.7)	10(25.0)	30(75.0)	17.2(4.5)	(15.8-18.6)	t_(174)_ = 8.0; ***P*** **< 0.001**
	Yes	136(77.3)	108(79.4)	28(20.6)	9.3(5.7)	(8.4-10.3)	
Partner/ parent reaction	Positive	101(57.4)	87(86.1)	14(13.9)	8.0(5.4)	(7.0-9.1)	(*F* (2, 173) = 40.85; ***P*** **< 0.001**)^†^
	Negative	36(20.5)	14(38.9)	22(61.1)	15.6(5.1)	(13.9-17.3)	
	Ambivalent	39(22.2)	17(43.6)	22(56.4)	15.0(5.2)	(13.3-16.7)	
Has other children	No	138(78.4)	88(63.8)	50(36.2)	12.0(6.2)	(11.0-13.1)	t_(174)_ = 3.7; ***P*** **< 0.001**
	Yes	38(21.6)	30(78.9)	8(21.1)	7.8(6.1)	(5.8-9.8)	
Experienced a stressful life event	No	53(30.1)	51(96.2)	2(3.8)	6.0(4.1)	(4.9-7.1)	t_(174)_ = −8.2; ***P*** **< 0.001**
	Yes	123(69.9)	67(54.5)	56(45.5)	13.3(5.9)	(12.3-14.4)	
Experience substance abuse	No	161(91.5)	112(69.6)	49(30.4)	10.8(6.3)	(9.8-11.8)	t_(174)_ = −2.4; ***P*** **= 0.019**
	Yes	15(8.5)	6(40.0)	9(60.0)	14.8(6.2)	(11.4-18.2)	
Attending clinic as scheduled	No	15(8.5)	1(6.7)	14(93.3)	18.7(3.4)	(16.8-20.6)	t_(174)_ = 5.2; ***P*** **< 0.001**
	Yes	161(91.5)	117(72.7)	44(27.3)	10.4(6.1)	(9.5-11.4)	
Experience domestic violence	No	108(61.4)	90(83.3)	18(16.7)	8.6(5.9)	(7.4-9.7)	t_(174)_ = −7.8; ***P*** **< 0.001**
	Yes	68(38.6)	28(41.2)	40(58.8)	15.2(4.8)	(14.0-16.3)	
Currently diagnosed with HIV/AIDS	No	162(92.0)	117(72.2)	45(27.8)	10.6(6.3)	(9.6-11.5)	t_(174)_ = −4.0; ***P*** **< 0.001**
	Yes	14(8.0)	1(7.1)	13(92.9)	17.4(3.3)	(15.5-19.4)	

*Note*:^†^One way Analysis of Variance (ANOVA); t-Independent samples t-test (Higher PHQ-9 mean scores translates to higher depression levels)

Not *Depressed*^*1*^*and Depressed*^*2*^is based on PHQ classification of 0-14 and 15 onwards. Mean scores and 95% CI are based on scores of depressed participants

### Depression prevalence

**Fig. 1:**
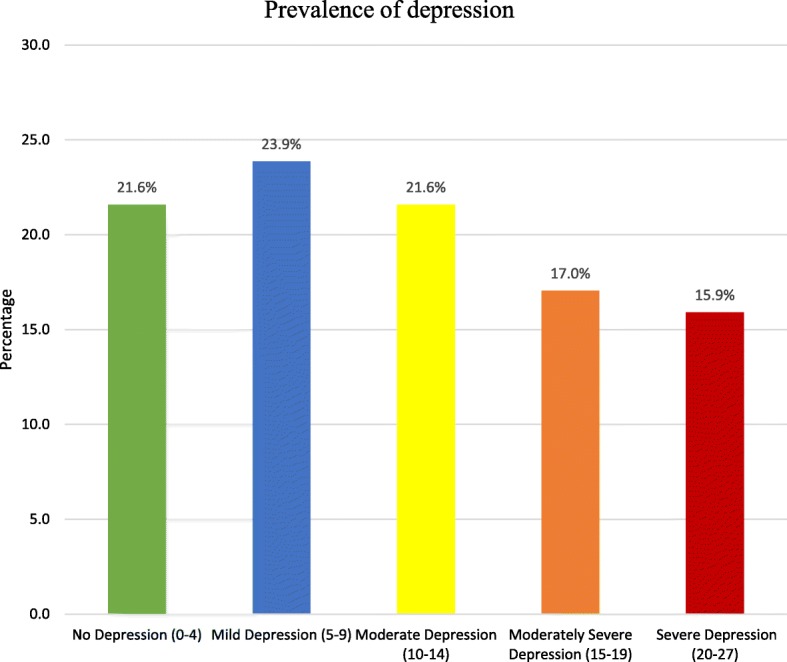
Depression Severity on PHQ-9. The figures in brackets denote PHQ-9 severity bands

The EPDS scale was used to screen the risk of depression in our pregnant adolescent sample and we found 58% falling under the category of a likelihood of depression. This was confirmed with the PHQ-9 diagnostic and severity assessment tool when a total of 33% (*n* = 58) tested positive for clinical depression. Out of 176 participants, 38 (21.6%) had no depression, 42 (23.9%) had mild depression, 38 (21.6%) had moderate depression, 30 (17%) had moderate severe depression while 28 participants (15.9%) were found to have severe depression (see Fig. 1).

### Associations between socio-demographic factors and adolescent depression

We used PHQ-9 continuous scores as our main outcome to test associations with socio-demographic factors. On *chi-square* test of association, the age of the participants was significantly associated with their depression status, *χ*^2^_(1, *N* = 176)_ = 8.84; *P* = 0.012. All of the 27 younger participants (ages 15-16) were depressed, as were the majority of older participants. Of the 176 pregnant adolescents of ages 15-18 years sampled in the study, 32.9% (*n* = 58) tested positive for a depression diagnosis using PHQ-9 cut-off of 15+. We detected a significant difference those who were depressed and not depressed using PHQ-9 cut off on educational level, marital status, living with parents, monthly income (where we found an inverse relationship, adolescents who were in households earning more than 15,000 kes/per month had higher depression, χ^2^_(1, *N* = 176)_ = 27.59; *P < 0.001*), housing type, receiving social support, experienced stressful event or substance abuse, attending ANC clinic, experiencing domestic violence and having a HIV positive status (see Table 1).

Univariate analyses (see, Tables 1 and 2) findings indicated that *younger age* (*F*(2, 173) = 18.63; *P* < 0.001), *unemployed* (*F* (1, 174) = 5.49; *P* = 0.020), single status t_(174)_ = 8.4; *P* < 0.001, *living with parents* t_(174)_ = − 8.1; *P* < 0.001, *higher monthly income* (*F* (2, 173) = 3.42; *P* = 0.035), *temporary housing* t_(174)_ = 4.7; *P* < 0.001, *not receiving social support* t_(174)_ = 8.0; *P* < 0.001, *ambivalent to negative reaction to pregnancy* (*F* (2, 173) = 40.85; *P* < 0.001), *having children* from before t_(174)_ = 3.7; *P* < 0.001, *experiencing a stressful event* t_(174)_ = − 8.2; *P* < 0.001, *those experiencing substance abuse* t_(174)_ = − 2.4; *P* = 0.019, *not attending clinic regularly* t_(174)_ = 5.2; *P* < 0.001, *those experiencing domestic violence* t_(174)_ = − 7.8; *P* < 0.001 and *HIV positive diagnosis* t_(174)_ = − 4.0; *P* < 0.001 were significantly associated with higher depressive scores.

**Table 2:** Results of hierarchical multiple regression analysis on depression

Variable	Category	Beta(S.E)	95%C.I Beta	β	*P*-Value	R^2^ change	*F-* ratio R^2^ change
Age in years	15 to 16 Years	2.46	(0.1-4.8)	0.14	*0.038*	0.372	12.3^a^
	17 Years	0.32	(−1.3-1.9)	0.02	0.701		
	18 years	*Reference*					
Occupation	Employed	0.11	(−1.7-1.9)	0.01	0.903		
	Unemployed	*Reference*					
Marital status	Married	0.00	(−2.6-2.6)	0. 00	0.999		
	Unmarried	*Reference*					
Living with parents	Yes	1.11	(−1.5-3.7)	0.09	0.403		
	No	*Reference*					
Type of housing	Permanent	−0.27	(−2.2-1.6)	−0.02	0.782		
	Temporary	*Reference*					
Income	< 10,000Ksh	0.27	(−1.7-2.3)	0.02	0.788		
	10,000-14,999	−1.04	(−3.0-0.9)	−0.08	0.286		
	> = 15,000Ksh	*Reference*					
Receiving social support	Yes	−2.76	(−4.8--0.7)	−0.19	*0.008*	0.181	7.1^a^
	No	*Reference*					
Partner reaction	Negative	1.46	(−1.2-4.1)	0.10	0.272		
	Ambivalent	0.96	(−1.4-3.4)	0.06	0.429		
	Positive	*Reference*					
Have other children	Yes	−1.58	(−3.4-0.2)	−0.11	0.089		
	No	*Reference*					
Experience a stressful life event	Yes	3.27	(1.4-5.1)	0.25	*0.001*		
	No	*Reference*					
Substance abuse	Yes	1.44	(−1.0-3.9)	0.07	0.246		
	No	*Reference*					
Experience domestic violence	Yes	0.99	(−0.9-2.8)	0.08	0.292		
	No	*Reference*					
Diagnosed with HIV/AIDS	Yes	3.81	(1.3-6.3)	0.17	*0.004*		
	No	*Reference*					
Whether attending clinic as scheduled	Yes	−2.55	(−5.2-0.1)	−0.11	0.059		
	No	*Reference*					
R^2^						0.554	

*Note*: ^a^Correlation is significant at the 0.01 level (2-tailed)

Beta –Unstandardized coefficient; β- Standardized coefficient

### Overall psychosocial risk factors model using multivariate linear regression

The results of multivariate linear regression using PHQ-9 scores as the dependent variable and 14 predictors in two blocks are shown in Table 2. The overall model with all the predictors were statistically significant and explained 55.4% of the variance in depression among the participants with *F* (17, 157) =11.46, *P* < 0.01. In Block 1, the respondents socio-demographic characteristics i.e. age, occupation, marital status, living arrangements and income explained 37.2% of the variance in depression scores, which was statistically significant with *F* (8, 166) = 12.31, *P* < 0.01), in which age, type of housing and marital status were statistically significant *P* < 0.05. In Block 2, other participants characteristics uniquely explained a statistically significant amount 18.1%, of the variance of the of participants depression after controlling for socio-demographical factors in Block 1, with *F* (9, 157) =7.10, *P* < 0.01. Therefore, the two blocks of variables significantly contributed to the prediction of the participant’s depression. After various iterations, when individual predictors using standardized beta scores were examined, *having experienced a stressful life event* (B = 3.27, *P* = 0.001, β =0.25), *the absence of social support for pregnant adolescents* (B = − 2.76, *P* = 0.008, β = − 0.19), *being diagnosed of HIV/AIDS* (B = 3.81, *P* = 0.004, β =0.17) and *being of younger age* (B = 2.46, *P* = 0.038, β =0.14) were the independent correlates of depression.

## Discussion

Our study findings that 32.5% of adolescents reported clinically elevated depression symptoms and that 15.9% of our sample had severe depressive features are in line with other SSA studies where prevalence of severe depression in pregnant adolescents is to be between 11 and 18% [9, 18, 43]. Our study identified four critical risk factors for depression such as *having experienced an adverse event or extremely stressful life context*, *living with HIV/AIDS, absence of support from the partner or the family* and *being a younger adolescent*. We found that 15.3% (*n* = 27) of pregnant adolescents were of ages 15-16 years indicative of a fairly early sexual initiation in our participants. The early sexual exposure with a concomitant lack of information/ knowledge or freedom to exercise sound sexual protection and contraception is a widely discussed issue in Kenya and in SSA in general. Doyle et al. (2012) in a study addressing the sexual behavior of adolescents in SSA reported that up to 25% of 15-19 years old’s across SSA are having sex before the age of 15 years [44]. Recent evidence suggests that young women are less likely to initiate sex if they are currently in school, with at least a secondary education and never married [9] and it is likely that pregnancy has resulted in dropping out of school for these adolescent girls who were between 15 and 16 years of age.

Other studies carried out in Kenya, Uganda and Ghana have pointed to an interplay of various social determinants associated with sexual and reproductive health such as poor education, high poverty and food insecurity, rigid social norms, and under-resourced health systems [9, 33, 45–48]. Given that our study was carried out in a health facility that caters to a large number of families based in informal settlements, each of these environmental factors play a mediating role that needs to be examined further.

We identified HIV positive status to be another risk factor in our study. We know that depression and stigma may impede linkage to HIV care, and to adherence to antiretroviral therapy (ART), additionally literature also suggests that ARVs and HIV positive status increases both HIV-related internalized stigma and depression [49–52]. We need adolescents to understand sexual and reproductive health decision-making much more clearly. Both HIV prevention and HIV treatment require basic skills in comprehending and digesting information (such as modes of transmission safe practices to avoid infection) and actioning it (learning life skills to negotiate safe sex practices with partners, avoiding sex to get material gains, and adhering to medication). We know that depression can impede that decision-making process [53] thus making adolescents more susceptible to high risk practices and behaviors.

The pathogenesis of depression is further magnified in the context of developing pregnancy in adolescents. A recent study on adverse life events and delinquent behaviors in adolescents from informal settlements on Kenya found that more than half of the adolescents lived in households characterized by either food insecurity or recent parental unemployment, and almost a fifth had dealt with multiple adverse events in their lives [54]. In such an environment where adolescent pregnancy means addition of another mouth to feed, the emotional, economic and social pressures multiply in the family leading to more internal conflicts and poor interpersonal relationships.

We found a negative correlation between presence of social support and depression status. Family and social support is a known protective mechanism in mitigating depression and mental health problems [23, 28]. However, given the research design we are unable to tease out the degree of dependence of these two variables.

Our study contributes to an understanding of how vulnerable pregnant adolescents living with HIV are to depression, social isolation consequently leading to poor utilization of health care and PMTCT services. We also interviewed a select group of participants and our survey findings tally with the lived experience of our participants mapped through the depression and psychosocial risk factors survey reported here [37]. Our findings suggest that the indicators for social support need to be multidimensional – covering parental, partner, educational and health contexts to assess the quality of support available to the young girls. Each one of these girls has unique familial, social and health realities that need to be accounted for in any psychosocial intervention development.

The strength of our work lies in the use of a robust depression diagnostic tool such as PHQ-9 to assess depression symptomatology further. Being a cross-sectional study carried out at a community health center in Nairobi, the study portrays a picture of depression, indicates severity and the association of probable major depressive disorder with psychosocial risk factors in the adolescent antenatal period. Despite addressing a gap in the literature around depression prevalence estimates for this sub-group of adolescents, our data is limited by design and cannot capture the causal directions of associations between the factors we studied and the same applies to the temporal sequence of development of depression in our adolescent participants. On hindsight, we would have liked to use more formal measures of social support, HIV related stigma, trauma and stress experience to identify the impact of these factors on our participants’ depression. Using a clinical interview to formalize the diagnoses of depression would have added greater strength to the study. A larger sample size would minimize any type 1 and 2 errors in estimating depression in this vulnerable population. Another limitation of study is that we collected data in a peri-urban health facility context and that may not sufficiently map onto the constraints and specific circumstances of pregnant adolescents in the rural or other remote settings in Kenya.

## Conclusion

Most evidence-based perinatal depression interventions in LMICs are focused on adults and there exists very limited understanding of depression related risk factors in depressed pregnant or parenting adolescents. Although a body of epidemiological research in adolescent pregnancies in LMIC context have established mental health problems to be dominant, for good intervention research vulnerable population specific risk factors need to be ascertained [4, 55, 56] and our study fills in that gap in depression prevalence estimate and associated risk factors in pregnant adolescents. We hope our study would provide pointers to critical factors that a depression intervention could encompass.

WHO’s Mental Health Treatment Gap Action Program (known as WHO mhGAP) [57, 58] argues for adoption of greater task-shifting models, low intensity and culturally adapted psychosocial models that would seek to inform mental health services implementation in resource constraint contexts. For pregnant adolescents in Kenya, we urgently need these components to be integrated in primary health care settings where we are likely to find young girls struggling with enormous social adversities and mental health challenges.

## Abbreviations

ANC: Antenatal clinic
ANOVA: Analysis of Variance
ART: Anti-retroviral therapy
EPDS: Edinburgh Postnatal Depression Score
ERC: Ethics and Review Committee
KNH: Kenyatta National Hospital
LMICs: Lower and Middle income Countries
MCH: Maternal and child health
PHQ-9: Patient Health Questionnaire-9
SD: Standard Deviation
SRH: Sexual and reproductive health
SSA: Sub-Saharan Africa
STIs: Sexually transmitted Infections
UoN: University of Nairobi
YLD: Years lived with disability
